# The Heroic and the Villainous: a qualitative study characterising the role models that shaped senior doctors’ professional identity

**DOI:** 10.1186/s12909-016-0731-0

**Published:** 2016-08-16

**Authors:** Kirsty Foster, Chris Roberts

**Affiliations:** 1 Medical Education and International Health, Sydney Medical School - Northern, Kolling Institute and Office for Global Health, University of Sydney, Level 7 Kolling Building, Royal North Shore Hospital, St Leonards, Sydney, New South Wales 2065 Australia; 2 Medical Education and Primary Care, Sydney Medical School- Northern, Kolling Institute, University of Sydney, Sydney, Australia

**Keywords:** Professional identity, Role model, Narrative analysis, Emotion, Clinical education, Reflection on behaviour

## Abstract

**Background:**

The successful development and sustaining of professional identity is critical to being a successful doctor. This study explores the enduring impact of significant early role models on the professional identity formation of senior doctors.

**Methods:**

Personal Interview Narratives were derived from the stories told by twelve senior doctors as they recalled accounts of people and events from the past that shaped their notions of being a doctor. Narrative inquiry methodology was used to explore and analyse video recording and transcript data from interviews.

**Results:**

Role models were frequently characterised as heroic, or villainous depending on whether they were perceived as good or bad influences respectively. The degree of sophistication in participants’ characterisations appeared to correspond with the stage of life of the participant at the time of the encounter. Heroes were characterised as attractive, altruistic, caring and clever, often in exaggerated terms. Conversely, villains were typically characterised as direct or covert bullies. Everyday events were surprisingly powerful, emotionally charged and persisted in participants’ memories much longer than expected. In particular, unresolved emotions dating from encounters where bullying behaviour had been witnessed or experienced were still apparent decades after the event.

**Conclusion:**

The characterisation of role models is an important part of the professional identity and socialisation of senior doctors. The enduring impact of what role models say and do means that all doctors, need to consistently reflect on how their own behaviour impacts the development of appropriate professional behaviours in both students and training doctors. This is especially important where problematic behaviours occur as, if not dealt with, they have the potential for long-lasting undesirable effects. The importance of small acts of caring in building a nurturing and supportive learning atmosphere at all stages of medical education cannot be underestimated.

**Electronic supplementary material:**

The online version of this article (doi:10.1186/s12909-016-0731-0) contains supplementary material, which is available to authorized users.

## Background

Along with the assimilation of knowledge and skills, the successful development of a medical professional identity is an important aspect of becoming a doctor. Modern conceptions of professionalism consider the underlying social and environmental forces that shape professional identity and behaviour [1] and internationally there is increasing research interest as to how medical education and postgraduate training shapes the professional attitudes and behaviours of both students and doctors.

Whilst role modelling is a powerful medium for the conveyance of professional behaviours [2–6] there are a number of gaps in the research exploring the processes by which this occurs. Most research investigates notions of professionalism from the perspective of medical student or junior doctor learners [7–17] through, for example, professional dilemmas [18, 19] or career choice [11, 20, 21]. This research investigates how experienced doctors perceive the impact of early medical role models on their own professional identity development and notions of professionalism.

In social science, the notion of identity is seen as a process of differentiating individuals and groups from other individuals and groups [22], It involves establishing similarity and differences between individuals or groups. Experiential workplace learning influences professional identity formation through a “two-way internal and external process whereby we define ourselves (who I think I am)” [23] and are simultaneously defined by others (who I think that you think I am) [23]. Similarities and differences between individuals and groups are established as part of the process of identification. In health professional education and practice, the terms ‘informal curriculum’ or ‘hidden curriculum’ are used to describe the often unintentional lessons learned from everyday encounters in healthcare settings [24–27] and operate at social and institutional levels [25, 27]). Concerns have been raised that the assimilation of this rich experience into evolving clinical practice has been neglected because of a prevailing focus on the formal curriculum, [28] the “stated, intended and formally offered and endorsed curriculum” [25, 29]

More recently the issue of “identity as a collective rather than an individually focussed undertaking” [20] has been highlighted as important for medical education. “Identifying ourselves, or others is a matter of meaning, and meaning always involves interaction” [22]. The way doctors behave as a group gives rise to the accepted professional norms which individuals entering the profession observe and learn. This process of 'occupational socialization' [30] contributes to development of a collective identity – in this case the accepted culture and mores of medicine as a profession.

The conceptual framework for this study is that, in the learning environment of the clinical setting medical students and junior doctors engage in complex interpersonal dynamics with patients and their families, doctors and other healthcare staff thus building their own professional identity through observing the behaviours of others. We explored the insights of senior doctors on the influences on the development of individual and collective identity of doctors from their own experience of becoming a medical professional as a way to illuminate understanding from a different perspective from the medical student and junior doctor perspective commonly reported in existing literature. Our assumption was that more senior doctors would offer a degree of reflexivity relevant to professionalism and professional identity not apparent in juniors with little experience of the profession.

## Methods

### Theoretical framework

The study is positioned in a constructionist epistemology, based on the tenet that meaning arises out of, or is constructed from, human interaction with the world and the things and people in it [31 p8]. Ontologically, constructionists believe that there are many ways of being, and that those multiple ‘realities’ depend on the interaction between people and things and upon social context [31 p42]. From a constructionist perspective there is no ‘true’ or ‘valid’ interpretation of the world as would be sought from an objectivist viewpoint but, rather, there are multiple credible interpretations [31 p48].

The broad theoretical perspective for this study is interpretivism, which fits well with a constructionist epistemology. The interpretive premise is that individuals construct meaning differently, depending on context, place and other circumstances. This theoretical perspective permitted consideration of the different layers and nuances of meaning in different people’s accounts of the concept of professional identity formation. The specific theoretical perspective for the research is symbolic interactionism, a perspective that looks at the way in which the actions and behaviours within a society are influenced by the shared meanings attributed to the words, gestures and other symbols that members of the society have grown up with and take for granted [32].

### Recruitment strategy

Recruitment to the study was via advertisements on a tertiary teaching hospital intranet site and in the local division of general practice newsletter. To broaden the range of specialties represented, purposive sampling [33] was done through an email to heads of specific departments not already represented. Any doctor qualified more than 10 years was eligible to take part in the study and all of those who volunteered participated.

### Data collection and analysis

The first author audio and video recorded in-depth, semi-structured interviews [34] which focused the participant on their early impressions of the medical profession and experiences which they felt had shaped their notion of professionalism. The interview guide was kept reasonably open ended in order to balance focus on the research topic with the exploratory nature of qualitative research. The guide is appended as Additional file 1. Memories were evoked, particularly related to professional education, training and practice and incidents (major or minor) sought from any stage in their life, which they felt had been formative to their developing notion of professionalism. Participants’ past encounters with clinical teachers were particularly explored because of the persuasive influence of role models on students’ and trainees’ professional development [6]. All interviews took place in a location chosen by the participant with most choosing their office or home. Interviews varied from thirty-three to sixty-two minutes in length with an average of forty-nine minutes. Each audiotape was transcribed by the first author and crosschecked with the videotape to ensure accuracy of content.

### Analysis

Narrative analysis was carried out in several stages using a dialogic performative approach [35]. Initially, a general thematic analysis focusing on the content of each interview as a whole, was conducted [35]. Secondly, small stories or personal incident narratives (PINs) within the data were identified using the definition “An event selected by the speaker as meaningful to the audience” [35]. Thirdly, thematic analysis of each PIN and a structural analysis focusing on the construction (or co-construction) of the narratives within the dialogic environment (see below) were conducted. Narrative inquiry allows abstract concepts constructed through the talk of research participants to become visible to the researcher. It recognises that “meaning is fluid, not fixed and universal” [36] and “‘explores the stories people live and tell” [37].

Narrative analysis has been used to explore the “what” and “how” of health professional students encounters in the workplace in which they witness or participate in something unprofessional [38, 39] revealing a complex interplay between identities, attribution of blame, and emotional residue. In our study, analysis of both the thematic and structural elements of the narratives (i.e the narrative process and content) of experienced doctors was used to elucidate the nature of professionalism within the culture of medicine as mediated through role models. It was anticipated that the senior doctor participants’ stories from their own past would be told from their current state of relative wisdom [40] and give insights into the impact of role models, and the informal curriculum during their training. This temporal dimension of narrative inquiry is well recognized [41] and adds depth to the interpretation. Through narrative analysis of the stories that people tell about their own experiences, it is possible to make a richer interpretation than would be possible from the content alone. The characterisation of the protagonists and the way in which the storyteller unfolds the plot contribute to meaning-making. These, along with the perspective adopted by the participant in telling the tale were carefully analysed and coded for each of the PINs [23].

At each stage the coding framework and emerging meanings were analysed individually and then negotiated between the two authors with a third colleague involved if discrepancies arose. In this way a complete picture of the influential factors on development of professional identity for this group of senior doctors was constructed.

Thematic and metaphorical analyses on the data supported the findings of the role model characterisation analysis. These results are not reported here because of lack of space.

### Ethical considerations

The main ethical considerations were maintaining the confidentiality of the participants and the possibility of emotional impact by reliving potentially distressing events from the past. The ethics committee was satisfied with arrangements made for keeping data confidential. Since an experienced general practitioner conducted the interviews and a protocol was developed for dealing with any distress arising, ethics approval was granted.

## Results

Twelve doctors were recruited. Nine participants were hospital-based, representing seven medical and surgical specialties (respiratory medicine, neonatal intensive care, accident and emergency medicine, obstetrics, gynaecology, paediatrics and psychiatry) and three were general practitioners. All were actively involved in teaching at undergraduate and postgraduate level. Two doctors were rurally based – one general practitioner and one hospital specialist. The range of time since qualification in medicine was 10 years to 40 years with roughly one third of the cohort in each decade group. All participants were Caucasian with nine graduates of three different Australian medical schools. The remaining three doctors had immigrated to Australia having trained overseas in Europe or New Zealand. These three doctors were all in hospital specialist positions.

One hundred and thirty-three personal incident narratives (PINs) were identified from twelve transcripts, which principally concerned interactions with doctors whom participants regarded as role models. Memories of the role models which doctors felt had influenced them were much more likely to be from their junior doctor experience rather than from medical student days. Almost half the stories were told from memories as an intern, junior medical officer, or registrar compared with only a quarter from medical student recollections. More than ten per cent of the stories were from childhood or schooldays all involving doctors who had been influential in an inspirational way. Stories chosen by all participants to demonstrate professionalism (or its lack) were about everyday encounters and interactions, which, although run of the mill, had endured in their memories over time. Critical events or ethical dilemmas, often used to teach professionalism [42], were rarely mentioned in any of the interviews. Characterisation of the protagonists in participants’ narratives fell into two broad groups: Those who were perceived to have been professional were constructed as “heroic”; and those who were remembered because of unpleasant experiences and associated with a lack of professionalism were constructed as “villainous”. Gender was a key component of the characterisations [43] and although heroines featured as well as heroes, villains were exclusively male. Furthermore heroes were perceived as having enabled or promoted learning while villains had inhibited learning. As is usual with rich qualitative data we have used typical examples to demonstrate emerging themes. Due to space limitations not all participants are quoted in this account. A summary of heroic and villainous qualities is given in Table 1.

**Table 1:** Characteristics of ‘Heroes’ and ‘Villains’ among medical role models

Hero (enables and promotes learning)	Villain (inhibits and prevents learning)
Shows and deals appropriately with emotion	Not able to deal with emotion – especially when bad things happen
Helpful	Bombastic
Supportive	Arrogant
Makes you reflect on own values	Dismissive of concerns
Good communicator	Hierarchical
Listens	Doesn’t listen
Able to interact with people	Elitist
Has high expectations	Directive
Flexible	Rigid / Inflexible
Gives praise (when due)	Intimidating
Good fun	Autocratic
Challenging (extends you)	Judgmental
Appreciates effort	Sexist
Gives autonomy in stages	Bullying
Left field – or not afraid to be individual	Unreasonable
Social	(Can be) highly skilled/knowledgeable
Highly skilled / knowledgeable/competent	
Respected and respectful	

### Heroic role models

Good role models were constructed as heroic in diverse ways. There was a common element of holding heroic characters in high regard, sometimes to an extraordinary and therefore “superhuman” level. Superlatives like “incredible”, “terrific” and “fantastic,” were commonly used in the narratives, especially those from childhood or from medical students days. These resonate with historical accounts of inspirational doctors of the past inspiring in students a sentiment “akin to hero worship” [44].

### Hero as outstanding clinician

Medical students are known to be idealistic about their chosen profession and about doctors [45]. Helena,^1^ a general practitioner, recalled a professor who had impressed her as a medical student by the speed at which he could accurately assess complex patient problems:

*And I felt that this guy knew his medicine and,....he could really clinch it [the diagnosis] in a few questions that you’d never even thought of. I was amazed by his clinical acumen*^2^

High intellect and great knowledge conferred heroic status [46] and Helena admitted to being ‘amazed’ by the professor’s intuition and ability to solve a case with only a few questions. She went on to become more effusive in her adulation of this teacher, employing powerful, almost poetic, organisation to her speech:

*and he just was passionate about medicine as well, so his professionalism was that he lived for the students, he lived for the residents he lived for the other consultants, he lived for education, lived for medicine. His passion was hard not to fall in love with. He knew everyone and that makes a real difference when a- when a senior teacher knows you by name and that is a big influence on you.” (Helena: 16 years previously)*

Here, Helena portrays the professor as someone who dedicated his life to his profession and to his students, and resonates with the iconic status of William Osler who over 100 years ago had attained the zenith of his reputation as a clinician of exquisite diagnostic capacity and human warmth, and a charismatic teacher who inspired a generation of students with a passion for the medical calling [47].

The repetition of the phrase ‘*he lived for’* is a potent and dramatic device suggesting extreme altruism and the word “passion” further emphasises the almost religious intensity to Helena’s perception of this particular role model. Overtones of infatuation in “hard not to fall in love with” suggest that Helena idolised this man. The last three lines of the PIN finally reveal that this professor, by knowing her name amongst all those that competed for his attention, had given legitimacy to her participation in medicine. This resonates with research showing how surgeons and students interpret and respond to each other’s behaviour, style, attitude and even demeanour, and the consequences for learning and teaching [48]. Our data suggests that positive role models are an important component of building legitimacy and trust which are central to learning and teaching in the clinical setting.

### Heroine as lovely

More than ten per cent of the stories originated from childhood. Molly, also a general practitioner, told a story about why she wanted to become a doctor.

*And I remember going through being a patient in casualty aged five and being looked after by a female probably Resident Medical Officer and thinking, particularly because she had lovely hair, that she was the most fantastic person in the whole wide world and that that’s what I was going to be because not only could you be a doctor but you could have lovely hair as well (Smiles and coyly moves head from side-to-side) (Molly: from 39 years previously)*

In recounting her story, Molly reverted to childlike language and voice as she recreated her 5-year old self. She imitated a fashion model showing off lovely hair as she emphasised the complex interplay between her multiple identities as first ‘woman’ and then ‘doctor‘ [20]. Girls are recognised to preferentially identify with role models of their own gender [49] and there is increasing recognition of the need to promote gender awareness in role modelling in medical education [50]. Molly’s story suggests that female doctors can inspire even very young female patients to aim at medicine as a career by appearing kind and well presented. Molly’s evolving professional identity appears to have been linked to her own socialisation into her gender-role, and her mental representation of what it meant to be a successful female doctor [51].

### Hero as adventurer

Derek, also a general practitioner, had been attracted to medicine as a career by a 1950s radio program:

*as a small boy at about the age of eight I decided I wanted to become a doctor. I was very much influenced by a doctor who’d worked in Africa. ....A man who had spent a number of years in East Africa working as a missionary doctor.... And in the fifties there was a program on the radio on Sunday afternoon that was called “Jungle Doctor” where he told many of the stories that surrounded his life as a doctor in East Africa. (Derek: 47 years previously)*

This PIN resonates with the heroic masculinity of a long passed Victorian cultural imagination of medicine and the essential disregard for the self [52]. Similarly, it speaks to a time of colonisation and the expansion of foreign missionaries. Many of the “Jungle Doctor” stories depicted African folklore and missionary adventure and included tales of good winning over evil. They told of surgical operations in the wild using the most basic equipment and quite often striking against the village witch doctors who relied on black magic to gain power.

### Hero as awe-inspiring

Ricky, now an academic surgeon close to retirement, told this story:

*And also at the end of my final year at school I came home from the second last paper in the [school] leaving certificate to see my mother being wheeled out of the house unconscious with a massive cerebral haemorrhage and these two guys [family doctors] were part of the team that cared for her and I just was in awe of the way they were always so supportive in every sense of the word and they appeared to be doing absolutely everything to both support us as a family but also to provide incredible care. (Ricky: from 44 years previously)*

In this PIN, Ricky tells the story from his perspective of being a schoolboy in the midst of final exams, who is suddenly faced with the trauma of his mother having a sudden and life-threatening illness. He portrays the doctors as heroes, relating how he was ‘in awe’ of them, that they provided ‘incredible care’ and emphasising that they were doing ‘absolutely everything.’ His personal experience of such remarkable care and support from doctors at this early and vulnerable stage in his life contributed to Ricky’s understanding of his sense of what it means to be a doctor [53]. In choosing to tell this story, Ricky interprets what was happening at the time in the light of what he now knows about the medical profession. In using his contemporary ‘voice’ as senior doctor he reveals something of his current view of professionalism, and one, which was shaped prior to medical school.

### Hero as teacher

Harry, now a rurally based hospital specialist, recalled the support he had from his supervisors when he worked overseas as a junior doctor

*And I think that’s where I enjoyed getting involved with, not particularly very sick [patients], but getting involved with [patients] day after day and the [relatives] and having a lot of autonomy, and a lot of responsibility and a lot of, and the [specialists] there were lovely men that were gentle and were from a rural background and that’s where it became very clear that that’s what I wanted to do. (Harry: 24 years previously)*

The atmosphere created in the telling of this story is calm and relaxed. Listening to the tape, Harry’s voice is gentle almost as if, even in the present, he is emulating the specialists he’s remembering from years previously. He talks about the enjoyment of being involved in patient care and keeps patients and responsibility central to the story. His heroes took an interest in him as a person and were courteous and polite. They took the trouble to find out what his abilities were and encouraged him to learn more by doing more. By valuing him as an active member of the team, they treated Harry with respect and gave him a safe space to develop his skills.

We would argue that the increasing sophistication of the characterisation of heroes in these narratives reflects various stages of the development of a professional identity and the complex socialisation into the profession of medicine.

### Villainous role models

A major element in forming their professional identity for our participants was encountering doctor role models whom they characterised as villains. The participants talk suggested an internal conversation across the years, with the villain’s part in the story clear and unchanged but the protagonist’s part unfinished. There is a lingering feeling of inadequacy, even guilt, that they had been powerless to stop what they considered to be extremes of unprofessional behaviour. There was a dissonance between on one side, feelings of anger and sadness at the deficiencies of a health system, which had left them (and patients) at the mercy of a villain and on the other knowing that a properly run health system should have dealt with such bullying behaviour.

### Villain as a bad clinician

Roy’s story about his experiences as an intern, many years earlier left little doubt about the strength of the feelings he felt at the time.

*Sometimes I wish I could have my RMO 1[second post-graduate year] over, or one day of it so I could tell one of the surgeons to fuck off. I wish I had and never have. He was incredibly awful, you know, I think his opening words to me when I started the term were “Are you going to cry? The last three have cried.” So - and I didn’t – but….. He was offensive to patients, very offensive to women and to registrars.*

Fifteen years later Roy still has significant emotional residue from his professional dilemma of 15 years previously [38] in that even now recounting the situation makes him angry. Roy’s characterisation of his supervisor develops into an aggressive villain from whom patients “had to be protected.”

*There was a very good registrar who actively hid patients from him because he wasn’t a very good surgeon and so, and I remember the hospital system dealt badly with him [the consultant] It was known amongst the junior doctors, this guy’s not a good surgeon, that patients had to be protected from him but it took a long time*

Interestingly, Roy does not project himself as the hero of the piece but casts the “very good registrar” as hero. This suggests an important role for middle-grade medical staff as an ameliorating influence in the informal curriculum for interns, as has been found in other studies [24]. The litany of unprofessional behaviours, with which Roy had a professional dilemma continued

*There was, you know, he assaulted one of the staff - I can’t remember who it was now, but he actually hit them so we were not empowered, not empowered as junior medical staff. There’s no way I’d tolerate it now. (Roy: 15 years previously)*

The vehemence of his feelings of anger and distress is clear from the words used as he relates the story of the encounter. He is angry with the surgeon who greeted him in a very inappropriate way at the start of a new job. He’s angry at himself for not standing up to the consultant at the time and he is angry at the ‘system’ for allowing the surgeon to behave in that way. He is also shocked and disillusioned. The realisation that doctors sometimes behave in less than ideal ways leads Roy to construct this doctor as an incompetent, uncaring and bullying villain; qualities quite the opposite of the heroic characterisations discussed earlier. Whilst others such as Julie are confident *villains such as Roy’s supervisor “just wouldn’t get away with that now”* (see below), Roy’s appreciation of the professionalism of others was tempered by his experience of how ‘the system’ at the time dealt (or not) with a poorly performing doctor. The system in Australia now would anticipate that a complaint about such a supervisor would be reported to the Australian Health Practitioners Registration Agency AHPRA and the National Medical Board may be able to do something to keep the public safe (https://www.ahpra.gov.au).

### Villain as teacher

Some features of Roy’s supervisor, bullying and intimidation are known to be prevalent in medicine [54]. Belittling of residents for example expecting them to break down and cry at some point is somehow regarded as a rite of passage into the profession [55].

The next PIN from Kevin constructs a bullying villain who he felt belittled him when he was a medical registrar:

*And when the older doctor came in he actually crossed out all my [patient management] plans and changed it to the way he wanted it to be. He said ‘You’re junior, you don’t know anything’. and I went to the books and I saw “wait a minute, I was right” Why didn’t he explain it to me? Why didn’t he explain to me why he changed it? –(Kevin: 7 years previously)*

Kevin creates a villain who fails in his supervisory duties by not explaining the reasons for changing the plans. His feelings of humiliation and of being excluded are emphasised by the use of direct speech and rhetorical questions in the narrative. Not only was Kevin excluded from discussion, which might have assisted his learning about patient management, but he also felt accused of knowing nothing and of being junior. This is a more covert type of bullying than the direct verbal and physical bullying in the previous PINs. The undermining of his professional identity damaged his relationship with his senior colleague at the time and may well have affected his relationships with subsequent supervisors.

### Villain as sexual harasser

Julie’s PIN about a bedside teaching session when she was a medical student presents a different type of villain. This incident took place with a group of twelve students being taught by a surgeon:

*I remember a patient one day, oh this was terrible. She had varicose veins and he [the surgeon] made her stand up on the bed and had us all looking at her legs, kind of looking, I thought “this is like we’re all peeping up her skirt.” And he said “If you were my wife I’d throw you out of bed for complaining about these veins” and then sort of slapped her on the …on the bottom and I …you looked …. he just wouldn’t get away with that now. (Julie: 22 years previously)*

Julie characterises the surgeon as using his position of power to sexualise and belittle the female patient in a patronising and patriarchal way. Julie not only uses direct speech to convey what the surgeon said to the patient but she also imitates the way in which he spoke to the lady. In the video Julie looks uncomfortable, almost as if she is reliving the way she felt all those years before. Witnessing the patient being “slapped” on the bottom, an aggressive act with sexual undertones was traumatic for Julie. Seeing a member of the medical profession behave in a way which was not acceptable in normal life, and seeing that he “got away with it” in his workplace left her speechless. In telling the story more than two decades later Julie stutters in a previously fast moving, fluent narrative almost as if she can hardly speak with the emotion evoked by the recollection of this behaviour. In the last sentence there is a strong statement that Julies feels that contemporary professional standards have changed as she brings us right up to the present and indicates that such behaviour would no longer be tolerated. Although it may be that modern curricula do little to prepare all students witnessing sexual harassment, with huge variation amongst students and teachers as to where thresholds lie when it comes to inappropriate behaviours in student–teacher encounters [56].

### Villains as a danger to the public

Trust is important in the doctor-patient relationship [57]. Lying to cover up mistakes where patient safety had been compromised was a common theme in the narratives. Alan told a story which had shocked him as a registrar when assisting a senior colleague at an outpatient clinic. A female patient had come back for review following a surgical procedure during childbirth. It was evident that one of the sponges used during the operation to mop up blood had been left in her vagina, a medical error, and one of which the patient was unaware.

*You know I remember some terrible things, like [surgeon’s name] left a sponge in someone after a repair and the patient came back a week later and complained about the smell and things and said she’d taken out this sponge and I was just stunned by his response to her. He said.” What? You didn’t take it out did you? I left that there intentionally I was going to take it out next time” (laughs) And that was his mechanism for dealing with it which was pretty quick thinking I have to admit but it was just the way people covered things. (Alan: Approximately 36 years previously)*

Alan introduces the story as describing “terrible things,” but in the end Alan is honest about his ethical ambivalence to this situation. His characterisation of this protagonist is almost as antihero (a person who does not display heroic qualities) rather than as villainous. He was stunned at the time by this behaviour. Whether by the way the surgeon lied so easily or by the brilliance of his rapid response is unclear. However, in the way this story is told there is a suggestion that the deviant behaviour was not only acceptable to the prevailing culture at the time but also worthy of admiration now. Not only was the surgeon lying to the patient, he was also making her feel foolish because she had taken the sponge out herself. Even so, Alan’s memory of this incident had persisted for almost 40 years. He seemed to be impressed by the quick thinking evident and appeared to condone the surgeon’s ‘covering up’ as a normal part of a successful career.

## Discussion

Interestingly there has been more written about characterisation of fictional role models than about real doctors going about their day to day work. Early medical heroes on television such as Dr. Kildare [58, 59] reinforced the positive public image of physicians, in a formula involving a hospital setting in which an attractive male physician of considerable biomedical acumen, along side his male mentor, assorted other doctors, nurses, and orderlies dramatically saved the lives of acutely ill patients [58, 59]. Nowadays, however, medical heroes, heroines, villains and villainesses are more complex. Modern villains can be constructed as those driven as the result of a harsh and unfortunate past resulting in the blurring of the boundaries between hero and villain. House MD, for example, is the opiate-addicted head of the “Department of Medical Diagnostics” at Princeton Plainsboro Hospital, who repeatedly flouts ethical rules and professional norms as he and his team relentlessly search for the correct diagnoses of patients’ symptoms [60]. There have been reports of concern about how such role model, albeit fictional, might adversely affect watching students’ and doctors’ professional behaviour [61].

A contemporary reading of these fictional stories inevitably set them against texts describing a spectrum of real life medical practitioner actions and behaviours from the systematically evil and the committing of serious medical error, through to the much more common notion of “lapses of professionalism” [18, 62].

Our findings reveal a myriad of relationships, hierarchies, power struggles and political agendas encountered during the development of professional identity through socialisation into the medical profession. We have illustrated some of the complexities around the phenomenon of role modelling and demonstrated that the shaping of senior doctors professional identity is multi-faceted, complex and often begins long before entering medical school.

Doctors in this study appeared to recognise professional lapses or dilemmas [18, 19] as they happened to them but the moral actions they proposed [38] in response to these dilemmas, increased in complexity along with their seniority and experience in the profession. Analysing emotional talk within narratives suggests that senior clinicians struggle many years later with the contradictory formal and informal learning experiences around professionalism, which resonates with the findings of others regarding the experiences of medical students [38, 39]. It was notable that memorable experiences often occurred in everyday routine and almost every narrative in this study involved informal learning within the hidden curriculum, [24–27] rather than the formal curriculum.

On the one hand the characterisations of role models included attractive, daring, clever, capable, selfless, enthusiastic, supportive, caring, encouraging and appreciative. On the other hand memorable characterisations of role models included bullying, abusive, exploitative, belittling, exclusive, sexist and dishonest. All experiences, good or bad, form part of socialisation into the culture and values of medicine as a profession and potentially contribute to professional identity formation [20].

### Implications for medical education

Our findings suggest that the major attribute of the senior doctors' heroes is that they *care* not only for their patients but also for their students or trainees, and that caring is fundamental to the professional identity of a doctor. Where caring for others, whether they are patients, family members or colleagues is the norm a hidden curriculum promoting care and compassion emerges.

Clinical educators and supervisors are often unaware of the powerful influence they can have on students and trainees [9]. This study shows that regular everyday interpersonal encounters and relationships are remembered and reflected upon many years after they occur. This is especially true if strong emotions have been evoked. Clinical educators need to be more aware of the impact of the things they say to juniors, particularly in emotive contexts and be more sensitive to the need to both give and receive feedback where there has been misinterpretation.

This rich symbolism of “heroes and villains” provides a mechanism through which both learners and their supervisors can become narrators to create, learn and pass on a more sophisticated reading of their culture [32]. Contact with both heroes and villains early in their careers gives new doctors a mixed and often conflicted impression of the values [18] and culture of medicine as a profession. A bullying and sexist environment was frequently described in the stories and this unsavoury aspect of medical culture has been raised again recently in the social media with allegations made against a senior by female junior doctors [63]. It is imperative for all members of the medical profession to tackle this situation head on, and recognise their individual contribution to the culture of medicine into which juniors are socialised. All doctors must take responsibility for maintaining the highest of professional standards themselves to ensure that students and trainees observing them see desirable professional behavioural norms in action and learn from them. The high regard with which doctors have in society [64, 65] is a cornerstone of doctors’ professional identity and brings with it a responsibility to ensure a collective professional identity worthy of such respect.

We believe our findings can be used in faculty development workshops as a practical way of encouraging clinical teachers to reflect on their professional identity both as a doctor and as a role model and to agree collectively how best to harness the power of the informal curriculum in their learning and teaching activities. Crucial elements of such workshops would be practical consideration of how supervisors may encourage open and meaningful discussion with trainees in the course of their education to highlight the priority of such issues and support development of a collective change. Using real examples of the lived experiences of students and junior doctors in which doctors are cast in the role of heroes and villains including the emotions evoked may help to bridge the gap between the idealism of the lecture theatre and the reality of the ward or clinic.

Professionalism dilemmas experienced by senior doctors, including issues concerning whistleblowing and challenging inappropriate behaviours, have implications for continuing professional development for all doctors. By focusing on common professionalism issues at a conceptual level, doctors can work through their real life experiences through sharing their stories and this is a model, which could be used widely. An additional possibility is that role-playing of idealised actions (how doctors wish they had acted) can enable doctors to commit and re-commit to professionalism values collaboratively [38]. The rich characterisations of medical heroes and villains on television [58, 61] also provide an opportunity to promote reflection on professional identity development for both medical students, doctors in training and senior doctors.

### Limitations of the study

Two of the participants were husband and wife working in different specialties in different hospitals which may have led to similar issues being raised in the two interviews. In fact they presented very different experiences and chain sampling where one participant ‘recruits’ another is well recognised in qualitative research especially where a particular group (in this case experienced doctors) is sought. Although the study was carried out with doctors from a broad array of specialties and age groups, all participants were Anglo-Celtic Australians, limiting the diversity. The relative ethnic homogeneity of the sample means that care needs to be taken in transferring the findings to other cultures. Within the culture of medicine however there is also diverse range of disciplines and roles that doctors undertake. One area for further research is whether there are differences in the cultural norms and behaviours accepted within the various professional sub groups of medicine.

## Conclusion

The characterisation of role models is an important part of the professional identity and socialisation of senior doctors. We have demonstrated the enduring impact of not only *what* these role models say and do, but also of *how* they speak to and treat people. This means that all doctors, need to consistently reflect on how their own behaviour impacts on the healthcare learning environment and consequently the development of professional behaviours and culture. This is especially important where problematic behaviours occur as, if not dealt with they have the potential for long-lasting undesirable effects not simply on the professionals involved but on the people they look after. The importance of small acts of caring such as using someone’s name, in building a nurturing and supportive learning atmosphere at all stages of medical education cannot be underestimated.

## Additional file

Additional file 1: Semi structured interview guide used in the study. (DOC 23 kb)

## Abbreviations

PIN: Personal Incident Narrative
RMO: Resident Medical Officer
AHPRA: Australian Health Practitioner Registration Agency
MD: Doctor of Medicine

## Footnotes

1 All names in this study are fictitious to preserve anonymity.

2 Narratives are presented with line breaks giving a rhythm to speech as in the way they were spoken.
