# Supplementary material for: The Heroic and the Villainous: a qualitative study characterising the role models that shaped senior doctors’ professional identity
Source: BMC Med Educ. 2016 Aug 16;16:206. doi: 10.1186/s12909-016-0731-0 (PMC4986406; doi:10.1186/s12909-016-0731-0)
Supplement: Additional file 1: — Semi structured interview guide used in the study. (DOC 23 kb) [file 12909_2016_731_MOESM1_ESM.doc]

**Semi structured interview guide used in the study**

After settling participant start with general warm up question to orientate to the topic

**1 Tell me what “professionalism” means to you as a doctor.**

2 Describe some of your early impressions of the medical profession from your early clinical training

**3 What can you remember of the hospital culture**

4 Were there any people who made a particular impression on you at that stage in your career?

Each of these will be explored in detail looking at the relationship to participant, characteristics which made them memorable, any particular events etc in detail

5 Can you recall any particular events during your early training which made you think about professional issues?

Each of these will be explored to discuss context, other people involved, and how the participant felt about them

**6 Did you enjoy your early clinical training? What aspects did you enjoy / not enjoy?**

**7 Do you think these early experiences influenced your own behaviour as a doctor?**

**If so , how?**

**8 Were there any other influences on your own development as a young professional which you think would be relevant to this study?**

Depending on the response to each question the interviewer will explore the answer in more depth and take any leads which emerge.
